# Overwhelmed by Technostress? Sensitive Archetypes and Effects in Times of Forced Digitalization

**DOI:** 10.3390/ijerph18084216

**Published:** 2021-04-16

**Authors:** Óscar. R. González-López, María Buenadicha-Mateos, M. Isabel Sánchez-Hernández

**Affiliations:** Business Organization and Sociology Department, School of Economics and Business Administration, University of Extremadura, 06006 Badajoz, Spain; orodrigo@unex.es (Ó.R.G.-L.); buenadic@unex.es (M.B.-M.)

**Keywords:** technostress, techno-anxiety, archetypes, social networks, digital economy

## Abstract

This paper explores technostress and its dimensions, assessing the relationship with possible negative effects in the individual, social and professional sphere. The study uses a self-reported approach of undergraduate students in Spain (*n* = 337), forced to follow their academic life by using technology comprehensively because of social distancing, as a public health action necessary to reduce the spread of COVID-19. The analysis, based on the exploration of a system of archetypes of the use of social networks, presents insights into contemporary technostress management as a new approach that can suppose opportunities for the optimization of prevention plans. Pearson’s correlation coefficients and structural equation modeling based on partial least squares (SEM-PLS) were the methods used for achieving the goals. The results reveal valid and reliable measures where technostress has a high impact on the individual sphere of students and there is a significant relationship between the type of user and techno-anxiety. The conclusions point to the imperative for developing a deeper understanding of technostress by archetypes, in both a higher education context (as antecedent) and the world of work, in an irreversible move towards a digital economy.

## 1. Introduction

The employment scene is rapidly changing because important transformations are reshaping the world of work, and many of them are related to the constant introduction of technology. It is evident that digital technology has opened up a huge range of possibilities in all modern economic systems [1]. Thanks to these rapid advances in technology, we can now speak of the fourth industrial revolution, which offers interesting changes in the way organizations operate, and the way people learn and work [2,3]. However, working conditions are also facing new challenges due to work technologies, including balancing work and private/family life, job stress interventions, or other aspects of the employees’ well-being [4]. Hence, there is a need to clarify how technologies influence work and employees.

It is a fact that information and communication technologies (ICTs) are being introduced in a vertiginous way in work and organizations [5] and educational contexts [6]. The benefits they can offer are numerous and relevant such as the management of large volumes of information with the minimum investment of time, increased productivity in processes, reduction of the importance of the physical and temporal dimension when carrying out tasks, favoring the possibilities of reconciling personal and work life, amongst others. This is not new and has been experimented with for some time now.

Currently, this has become a topic of even greater interest as a result of the COVID-19 pandemic: the remarkable growth in digitization has occurred to respond to situations that have been affected by the virus at a personal, social and organizational level. For example, data from the Spain Digital Plan 2025 report [7] reflect that the pandemic has boosted and accelerated digitization, with increases in Spain in mobile data traffic (compared to 2019 of 50%). Internationally, 97% of business executives believe that the pandemic accelerated the digital transformation of their company, and one out of three companies started using live chat and interactive voice response (IVR) channels for the first time as a result of COVID-19 [8].

However, both the effects and risks of the introduction and acceleration in the use of technological resources in organizations play an important role in employees’ health, and therefore, in the proper functioning of the organization. For example, when individuals perceive that the use of technology is very high, they are more likely to feel stress, which has consequences in the different spheres of life [9]. In the same line, Fernandez-Ferrin et al. [10] have highlighted that the “accelerated digital adaptation has been imposed on educational environments, placing social inequalities and reconciliation of family and professional life at the center of debate” (p. 5). Thus, specific studies of these risks must be carried out to help ensure that the introduction of technology has the least negative impact on people.

Expressly, this work studies technostress, which is an emerging theoretical construct that has emerged from multiple currents of thought that still do not allow a clear vision of its scope and relevance [11], and due to the current digital intensification, it is increasing in importance in the working world. After this introduction, the paper explores technostress and its dimensions, assessing the relationship with possible effects in the individual, social and professional sphere. Later, the paper presents the results of an empirical study, based on the exploration of a system of archetypes of use of social networks, of undergraduate students forced to social distance and to use technology. Finally, the discussion and conclusion offer a reflection on contemporary technostress management as a new approach that can suppose opportunities for the optimization of prevention plans.

## 2. Theoretical Background

### 2.1. The Personal/Professional Use of Technology

Technology profoundly impacts societal progress and economic development [12]. ICTs such as the Internet, advanced wireless technologies and mobile communication networks are increasingly indispensable in many aspects of daily life and business [13]. In fact, in many cases, the most valuable contribution of computers is not only the contribution of artificial intelligence but their ability to provide hyper-connectivity, managing to connect people in new and rich ways [14].

In recent years, we are observing an important transformation in the world of work. The new labor dynamics imply greater demands on people, since it is increasingly expected that employees achieve greater and faster production [11]. Technology at work promotes progressive changes in organizations. In order to improve productivity and efficiency, different technological innovations have been introduced that reduce processes and production time, improve quality, improve communication and in many cases, reduce occupational risks [15]. In addition, new needs, new organizations and new jobs have been created. In fact, some studies indicate that many people in the future will not need to work, at least in the way we continue thinking about human work [16]. Today, increasingly complex tasks can be automated with a precision that seemed unfeasible just a few years ago. Examples range from voice and image recognition to autonomous vehicles [17].

However, technology at work also has a dark side [18]. There are studies that link the introduction of technology with future job losses, since, at some levels, the labor market has changed and due to the implementation of new technologies, the operational workforce is being replaced by machines [19]. It is important to note that not only the operational level is affected. For example, artificial intelligence has penetrated many organizational processes, which is also generating a growing fear that machines may soon also replace humans in decision-making [20]. In fact, the next wave of automation technologies may accelerate the pace of change, causing multitudes of jobs to gradually disappear and new ones to be created [21]. In addition to all of the above, it should be noted that ICT also affects more specific aspects of work and the day-to-day nature of work could change for almost everyone as smart machines become accessories in the workplace [21].

Studies related to ICTs in organizations are of great importance, since these technologies (especially the use of mobile phones and social networks) are not only used in the labor dimension. They are also very present in the personal sphere of the workers. This is a unique and differential evidence compared to other technological introductions. For example, there are studies that show that the use of ICTs outside of workdays blurs work and non-work limits [22]. At this respect, it is remarkable that the most common use of the internet is through social networks. They are used to communicate, share, collaborate, and connect [23] and they have become some of the most powerful communication tools both inside and outside the workplace [24]. Specifically, some authors suggest that university students are more willing than others to develop a problematic use of social networks [25].

Much of the research on social networks and organizations has focused on their possible negative and destructive consequences at work [24] because, just as with smart phones, social media misuse can promote problems for employees. Overdependence on such media could, in some cases, be attributed to emotional escapism (from stress, depression, and other real-life problems) [26]. It is relevant that the exhaustion of social networks significantly reduces individual work performance [27,28]. In addition, the transformation of work in a globalized way, in which the “eternally available worker” appears, propitiated by the use of new technologies, makes working people more likely to get sick due to stress and technostress. Thus, technostress is associated with the use of personal social networks at work and a negative effect on performance [29]. Specifically, there are studies that show that the excessive use of mobiles induces burnout [30], has consequences on physical [31] and mental health [31,32,33] and is becoming a problem of public health [27,32]. There is also evidence that the compulsive use of smartphones is positively related to psychological traits of users [28]. There are also studies that use the term overload of social networks [25]. It can be said that the excessive use of social networks is a determinant of three types of overload: information, communication and social. In this sense, information and communication overload are important stressors that influence the depletion of social networks [27].

This double dimension of use—personal/professional—can offer both positive and negative consequences for users. Therefore, it is important to make a balanced review of the assessment of the benefits and possible disadvantages of the use of these technologies, which must be properly managed to maximize their positive effects on people. As Malone [14] points out, it is not likely that the most important uses of new technologies are to replace humans, but rather to allow people and computers to do better together than anyone could do alone. On the one hand, some positive aspects offered by ICTs are favoring an increase in motivation [34], a decrease in cynicism and a rise in levels of self-confidence [35], access to information in a very easy and fast way, simultaneous social contact and performance improvement [36]. On the other hand, and usually when the use is excessive, some negative aspects of technology must be taken into account—above all, the ones related to favoring possible isolation. Some authors highlight that machines or devices have hindered human relationships, isolating people and subjecting them to the constant use of mobile devices and multiple screens linked to different content [37]. Furthermore, the use of ICTs can promote instability in the worker’s family–work balance [38]. Other negative effects are related to pressure and stress. The pressure to finish work on time is one of the characteristics of the current labor market [39]. ICTs can also push employees to constantly connect to information systems and respond, almost immediately, to the demands of their supervisors [40] and there are studies that indicate that the invasive use of ICTs promotes greater stress in the workplace [38].

All these important changes generate the need to address the consequences of technological innovations in companies in order to prevent their impact at the individual, group and organizational level [41]. Consequently, all these changes require prevention and advice to avoid damage and unwanted negative effects of the technological impact on both the efficacy of companies and the psychosocial needs of workers. The dramatic irruption of virtual work during the COVID-19 pandemic will increase the number of employees working remotely [42], justifying the study of technostress.

### 2.2. Technostress, Sources and Effects

To keep up with the rapid advancement of new ICTs, employees must constantly renew their technical skills, as well as withstand the constant pressure of a more complex system and higher productivity expectations. This often leads to ICT-related technical stress, experienced by employees in many organizations [13].

The term technostress was coined by Brod [43,44] and defined as a modern adaptive disease, caused by the lack of ability to deal with new technologies in a healthy way. It is directly related to the negative psychosocial effects of the use of ICTs. Research on technostress has identified several sources of stress, such as the invasion of privacy, interruptions, or information overload, amongst others [18,45,46,47]. Tarafdar et al. [45] have identified five creators of technostress: techno-overload, occurring when the technology forces the employee to work faster; techno-invasion, related to pervasive ICTs invading personal life; techno-complexity, because of feelings of incompetence, as a consequence of the complexity of new ICTs; techno-insecurity, because employees feel that their jobs are at risk when ICTs change so fast; techno-uncertainty, because employees do not know what the following innovation will be. Job demands and a lack of both job and personal resources are related to technostress in anxiety, fatigue, skepticism and inefficacy [41], these four dimensions being relevant for conducting technostress studies. At this respect, some studies on technostress are being published in the COVID-19 context [48,49,50].

Moving to the effects of technostress, the academic literature group them into individual, group and professional/organizational levels [35]. There seem to be relationships between each level. For example, the consequences of the individual sphere may have repercussions on the group and/or organizational ones. Therefore, the study of these relationships is relevant since employers must play an important role in providing practices in the workplace that protect the physical and psychological well-being of employees [51].

#### 2.2.1. Individual Effects of Technostress

Two psychological experiences associated with the use of ICTs should be taken into account: techno-addiction and techno-strain [41]. With techno-addiction, users feel bad due to the excessive and compulsive use of ICTs. With techno-strain, users are overwhelmed and show a combination of anxiety, beliefs of ineffectiveness, fatigue and skepticism related to the use of technologies.

In general terms, technostress can have different individual consequences. These can be physical consequences (since technostress can have negative consequences on the health of those who suffer from it, being able to favor, among others, headaches, muscle aches, and gastrointestinal disorders), problems related to adequate rest favoring difficulty sleeping and monitoring irregular sleep patterns and can generate erratic dietary behavior [26] that in the medium term can lead to serious problems in the body. All these problems can have repercussions in other spheres.

#### 2.2.2. Group Effects of Technostress

Technostress can have social consequences. They can favor negative experiences in family life [52], and the excessive use of technology can lead to reduced face-to-face communication and privacy-related problems [26].

#### 2.2.3. Professional Effects of Technostress

Technostress can have professional consequences. Technostress is related to burnout [53]. Specifically, there are studies showing that technostress offers lower professional satisfaction with work [54]. It is undoubtedly important to highlight the significant negative impact of technostress on absenteeism and employee productivity [45].

Taking into account all of the above, it could be taken for granted that technostress has a direct and negative impact on overwhelmed users of technology at the individual, group and professional level, although it must be verified whether it is significant. Considering of all the above, and in the current context of forced digitization, we propose the first working hypothesis:

**Hypothesis** **1** **(H1).** *Technostress impacts the individual, group and professional sphere of overwhelmed users.*

### 2.3. Individual Differences

Progress in ICTs is influencing the development of human resource management (HRM) [35] in very diverse areas, such as employee recruitment (online recruitment and selection models), training or the transformation of internal communication [55,56]. Employers must consider the effects of ICTs to try to minimize the possible adverse effects on employees. Improving positive work experiences and promoting employees’ work commitment is a responsibility of the organization towards public health, which is even more important in modern times than before [57]. In this context, it is necessary to pay attention to technostress to focus on the necessary prevention and coping strategies.

Considering that stressors do not act or affect all workers in the same way, individual differences, such as the psychological traits of the users [28], their level of extraversion [58], personality [59], or age [60,61,62], could play a very important role in the perception of stress.

Some authors suggest that technostress depends on the psychological traits of users [28]. The proof is that one of the main problems posed by technostress as a risk for workers is the difficulty of measurement. As the subjective factor of the personality of the exposed worker is implicit, the same risk factor does not offer the same result in all people. For instance, technostress can affect workers of different ages, although the different capacities to work with ICTs require specific studies such as the work of Nimrod [61], focused on technostress in people over 60 years of age and showing that technostress as a particular threat for well-being in adulthood that can be very relevant especially in cases in which there is an aging workforce [60]. Despite having another relationship with new technologies, technostress among university students with the use of ICTs can also incur problems such as exhaustion, decreased learning commitment, reduced performance, and intentions to quit [61].

More specifically, and related to technostress, there are different behaviors in the use of the internet and social networks. These individualities could be a mediator in which the user experiences certain levels of technostress and certain consequences. The use of archetypes supposes a simplification of reality that entails a certain loss of information that, at the same time, facilitates the personalization processes in different disciplines of business administration (consumer segmentation, classification of employees according to their performance or personality type for recruitment and selection). Specifically, if it is about individual archetypes; they allow identifying the personal behavior of an individual and their development in the place where they are, through the qualities that define them.

Given the object of study of this research, it would be relevant to know individual differences in technostress and in the negative effects that were related to the way of using technology. There may be types of behavior in the use of social networks that serve as conditioning factors in suffering from different levels of technostress and suffering negative consequences at different levels. There is a typology that, even though it was developed in the context of the problematic use of social networks, establishes five archetypes of user behavior [63]. They are based on the internal characteristics of the users, emotions, and take into account the psychological states experienced in the participation in social networks and whose characteristics could also allow to classify users of less intensity (not problematic). A brief description of the behavioral archetypes is the following [63]:

- Archetype Secure: likes to feel assured and social media helps to maintain the feeling of security by building successful relationships that increase their social presence and connectedness.
- Archetype Intimate: has a fear of missing out and likes friends expressing interest and providing emotional responses.
- Archetype Escapist: does not like their reality and social media helps to live an invented parallel reality.
- Archetype Narcissist: likes to feel accepted by others and uses social media for competition with others.
- Archetype Discrepancy: is completely engaged in social media but is discrepant with the waste of time that this requires.

These archetypes not only form a solid basis for classifying social network users (with the aim of using intervention tools), but they can also differentiate types of users in other areas still to be defined through research and it can be useful in any organization setting. Therefore, we consider it important to investigate whether the type of user is related to the level of technostress, since this could focus its preventive and coping actions. In the same line of thinking, it is worth asking if the type of user is related to the adverse effects that can be experienced at the individual, group, and organizational level. Taking this typology into account, we propose the following additional working hypotheses:

**Hypothesis** **2** **(H2).** *Technostress depends on the psychological traits of users of social networks.*

**Hypothesis** **3** **(H3).** *There is a relationship between the different archetypes of use of social networks and the consequences of technostress.*

## 3. Materials and Methods

### 3.1. Participants

The sample selected for this work consisted of students from the School of Economic and Business Sciences at the University of Extremadura, Spain, during the 2019/2020 academic year. All participants were required to have lived the experience of forced transformation (due to the lockdown measures imposed by the pandemic) to online training and assessment of at least four subjects. This means that they have been subjected to intensive digital acceleration, both for the fulfillment of their academic responsibilities and for their personal and social life. All these students are close to their incorporation into the world of work in areas related to management information, which is why ICTs are of great importance.

A total of 337 students voluntarily participated in this study. A total of 61.13% of the sample were women and 38.87% were men. In relation to age, 1.78% belong to Generation X, 33.23% to Generation M and 64.99% to Generation Z (so the majority, 98.22 of the sample, belongs to MYZ generations). The most widely used device as a platform for internet use is the mobile (smartphone). Measuring intensity of use from 1 to 5, the mean mobile use is 4.87 (SD = 0.44). The most frequently used social network is WhatsApp ((4.84) SD = 0.44), followed by Instagram ((4.54) SD = 0.83) (measuring intensity of use from 1 to 5). In vital technological synchronization, the study participants, in an assessment of 1 to 5, indicated that their use of the internet in the first 5 min of being awake is on average 4.1 (SD = 1.11) and in the 5 min just before falling asleep (mean 4.59 (SD = 0.75)).

The fact that the participants were students has been considered interesting since the new information technologies also have an impact on the increasing levels of academic stress as a result of the pressure exerted by the environment to be updated and the excessive use of ICTs. In this respect, in the work of Barber and Santuzzi [64] a measure of general tele-pressure was validated in a sample of university students and they show psychometric properties similar to the original measure of the workplace.

### 3.2. Design, Procedure and Methods

On the one hand, this study raises the question of whether there is a positive relationship between the level of technostress and possible negative consequences at the individual, group and professional level, as well as whether there is a relationship between its dimensions. On the other hand, the typology of the archetypes of use of social networks by Altuwairiqi et al. [63] has been selected to study whether there is a relationship between these archetypes and the level of technostress, as well as between the possible negative consequences they experience at the individual, social and professional level. To achieve these exploratory goals, this study used a quantitative research method with the traditional Pearson’s correlation approach but complemented with structural equation modeling (SEM), based on partial least squares (PLS), using the software IBM SPSS Statistics 25 (IBM Corp., Armonk, NY, USA), and SmartPLS 2.0 M3 (SmartPLS GmbH, Bönningstedt, Germany). SEM-PLS, as a powerful second-generation technique, allows the definition and validation of a model for capturing causality and consequence in the hypothesized correlations.

**Figure 1:**
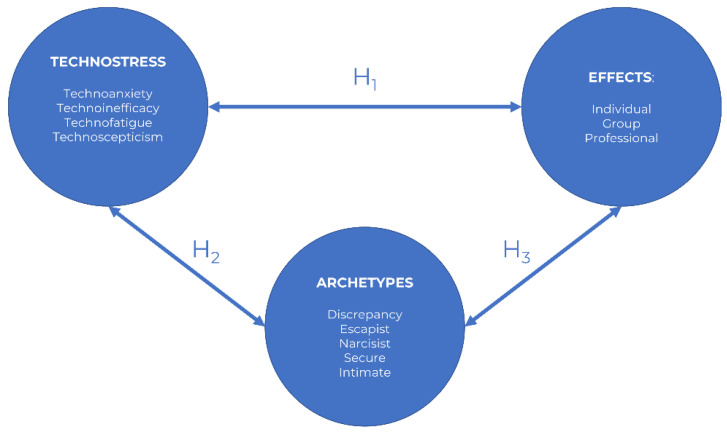
Research design.

Figure 1 shows a diagram reflecting the research design for a better comprehension. Table 1 shows the basis of the instrument developed for a self-reported perception of the selected sample of undergraduate students.

According to the literature review, the dimensions of technostress as a frame of reference were considered: anxiety, ineffectiveness of beliefs, fatigue, and skepticism, and related to the use of technologies that can be measured with the RED General Questionnaire [65]. The different negative consequences have been approached from those proposed by Cham et al. [26]. This work of digital addiction has been considered, grouping the items according to the dimensions of the effects of technostress at the individual, group and organizational level [26]. Regarding the archetypes of behavior on the internet, those raised by Altuwairiqi et al. [63] have been used.

The questionnaire was designed from the theoretical review and the measurement instrument was developed by the researchers to address the specific objectives of the study and consisted of four parts and a total of 30 questions.

- The first part (five items) refers to contextual aspects related to the sample.
- The second part (16 items) refers to the perceived experience of technostress, four for each dimension (skepticism, fatigue, anxiety, and ineffectiveness), grading its importance using a five-point Likert scale (from totally disagree to totally agree).
- The third part (eight items) refers to the negative consequences experienced by using the internet in different areas using a five-point Likert scale.
- The fourth part (one item) refers to the archetype of self-identification in the use of social networks (choose one of the five established types) described by the authors who carried out the classification.

A self-administered questionnaire designed ad hoc was used for this research; “Google Forms” from “Google Drive” was used for its application. The address of the questionnaire, as well as an access QR code, was made available to the students. The questionnaires were completed by the students during the 2019 to 2020 academic year. The questionnaire explained the exploratory objectives of the study, and the voluntary, confidential and anonymous nature of the responses. Collaborating students were awarded an extra-credit score of 0.1 in the final mark of the subject for their collaboration. During the data collection process, the subjects had the advice of an investigator and, previously, they were informed about the study and the meaning of the different parts. The data collected does not allow the identification of the participants in the research, thus complying with ethical recommendations and the regulation of personal data.

Participants were asked for their consent to participate in the study so we could extract and use this information for the sole and exclusive purpose of the research on technostress. To achieve this, a mandatory question that appeared in the first part (before viewing the specific questions of the questionnaire) was introduced. If this question was not answered affirmatively, access to the questionnaire would not be allowed. Once they had given us their consent to participate, the participants answered questions about their perceptions about the use of technology, about the frequency with which they have had problems in different dimensions by cause of being on the internet and general information about the study and data. In addition, people were asked to read each of the five archetypes of use of social networks and choose the profile with which they predominantly identified.

All data for this study were collected in Spanish. To ensure cross-cultural equivalence of the scales and the descriptions of the archetypes that were not originally in Spanish, two procedures were followed. First, to ensure the accuracy of the translation, the authors translated the scales from English into Spanish, and someone outside the study was asked to translate them into English. The authors reviewed the reverse translation and made the changes to items that could be interpreted differently. Second, a pilot study with a sample was undertaken to assess the reliability of the scales after translation. Based on this pilot study, several elements were reviewed before the final questionnaire was proposed. A correlation analysis was performed using Spearman’s correlation coefficient and Pearson’s correlation coefficient, which measure a linear relationship.

## 4. Results

### 4.1. Measurement Model

Regarding technostress, valued with scores from 1 to 5, and considering the means, the highest dimension is techno-skepticism, being the only dimension where the maximum was reached in any observation. Techno-skepticism, although it is the maximum value, was very close to techno-fatigue, which had the second highest value. Regarding the dimension with the lowest incidence in our study, it is techno-efficacy. The average valuation of technostress in general is 2.2 (SD = 0.69). Table 2 shows descriptive statistics of technostress and its dimensions.

The correlations of the different dimensions of technostress, as can be seen, have a positive and significant relationship with each other, and they all have a positive and significant relation with the global technostress indicator. The order from highest to lowest relationship of each dimension with global technostress is: techno-anxiety, techno-inefficacy, techno-fatigue, and techno-skepticism (Table 3).

Table 4 shows descriptive statistics of negative effects by dimensions. The results show that technostress has greater consequences at the individual level (related to emotional, personal aspects, damages and problems related to food), followed in importance by the consequences at the group level (related to social effects on family life, and privacy issues) and followed in importance by professional consequences (aspects related to performance).

Regarding the individual differences, Table 5 shows the composition of the sample by archetypes. As can be seen in the table, the most common archetypes are secure (34.7%) and intimate (31.5%). More than half of the participants would be contained in these two profiles. The least frequent archetype is the narcissist archetype (below 10%)

**Figure 2:**
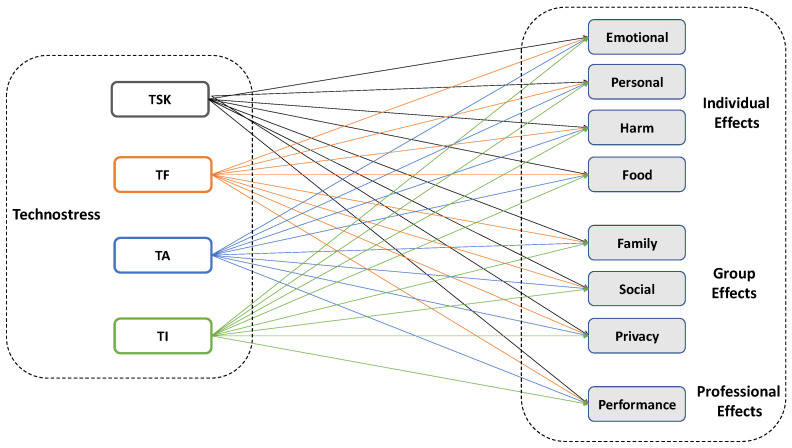
Theoretical cause–effect framework.

As a complement of the analysis, the model shown in Figure 2, represents the expected causal relationships between the constructs of this study focusing the attention on dimensions. A total of 32 potential causal paths are laid out, combining the four dimensions of technostress with the eight potential effects. Considering that it is a reflective measurement model—because, theoretically, items must be correlated—the reliability and validity of the constructs have been analyzed with SEM-PLS [66] (Table 6). Outer loadings between 0.60 and 0.94, composite reliability for all constructs above 0.70 showing consistency, and values for AVE greater than 0.50, confirm the quality of the measurement model being evaluated [67,68].

### 4.2. Hypothesis Testing

Regarding hypothesis 1, global technostress has a positive and statistically significant relationship with all three dimensions of consequences (individual, group and professional). The strongest relationship of global technostress occurs with individual consequences, followed by group ones, and the one with the lowest intensity with professional consequences. This ranking of importance also occurs when doing an analysis by dimensions, except in the case of techno-skepticism (whose ranking of importance is above all with the group, followed by the individual, and has no statistically significant relationship with the professionals).

It is essential to analyze the relationship between the different dimensions of consequences studied, since the initial idea that some dimensions are related to others must be contrasted. To do this, we studied Pearson’s correlations, as shown in Table 7 and Table 8. Therefore, we can conclude that indeed, all three dimensions of the consequences are related to each other in a statistically significant way, with the strongest relationships between individual and group consequences (0.74), followed by the relation between individual and professional consequences (0.66), followed lastly by those between group and professional consequences (0.48).

Moving to assess the direct causal relations between the constructs in the theoretical model, Table 9 shows the results obtained after the bootstrapping procedure based on 5000 iterations in SEM-PLS [69] confirming a significant relationship in most of the structural paths.

Moving to hypothesis 2, related to the archetypes and technostress relationship, as can be seen in Table 10, there is no correlation between the studied archetypes and the level of global technostress. Nonetheless, if we do the analysis by dimensions, we do find a positive correlation with techno-anxiety.

One of the objectives of this work is to know if there are archetypes more sensitive to technostress than others; studying the levels of technostress by archetypes, it can be observed in Table 11 that the highest global technostress is experienced by discrepancy, and the lowest by those of the intimate profile.

If we look at techno-anxiety (the only dimension with a statistically significant correlation with the archetypes) the order of the level of techno-anxiety by archetype, from highest to lowest, is: discrepancy, escapist, narcissist, secure and intimate) and that is exactly the hierarchy that the global technostress level has. In fact, the intimate archetype is the one with the lowest level of technostress, whether the global technostress is analyzed as if it is done by dimensions.

Finally, and related to hypothesis 3, as it can be seen in Table 12, the archetype correlates in a statistically significant and positive way with individual, group and professional effects. The highest correlation is with individual effects, followed by professional and group effects.

Analyzing the levels by archetype based on the means (Table 13), it can be seen that in the three dimensions, the narcissist profile is the one that suffers the most important consequences and those of secure and intimate the least, making it clear that in this case (as in the levels of technostress), the intimate profile is the softest and most desirable one.

## 5. Discussion

One of the biggest challenges we face in these times of digital acceleration is enabling people to maximize the possibilities that ICTs offer, in personal and professional life, without putting health at risk. This dual use makes it easier to assimilate the technology, but it can also create overload. Due to forced social distancing measures to avoid the contagion of COVID-19, the use of ICTs has increased, and it is more important than ever to investigate technostress [70].

Physical and mental conditions significantly influence the general health of people, and their daily performance [71] and one of the priorities that companies indicate in times of a pandemic is to attend to the physical and mental health of workers.

In the case of this study, a crucial and exceptional moment of intensive use of technology has been chosen, altogether at the individual, group and professional level. Knowing the risks of technostress, and assessing their effects, will help society to enable the management of preventive measures; facilitating the intensive use of ICTs leaves fewer health problems. Therefore, the purpose of this article was to explore technostress and its dimensions, and to assess the impact on the subjects. The study focused the attention on university students who were forced to follow their assignments, activities and assessments using technology comprehensively.

To achieve the aims of the study, the analyses have been based on the study of the possible correlations and cause–effect relationships between the selected constructs derived from a previous theoretical review.

Related to technostress, it has been proven that there is a relationship between its dimensions and between each of them. From highest to lowest incidence, techno-skepticism is the more relevant. In this sense, it does not match with the study by Estrada et al. [62] on technostress in teachers, where techno-anxiety is ahead of techno-fatigue, although with very similar values.

Techno-skepticism is attitudinal and manifests itself in cynicism and shows exhaustion. Those who suffer from it feel discouraged, as well as distant, and indifferent towards technology. In our study, it is interesting to observe that skepticism behaves differently from other dimensions, mainly impacting on the group, instead of having individual effects, as occurs in the other three dimensions.

Regarding the potential negative consequences of technostress, this study shows that there is a statistically significant cause–effect relationship between each dimension and the three levels considered. The hierarchy of relationships reveals that it is greater with individual, group, and lastly, professional consequences. It has also been empirically verified in this work that there is a relationship between the three dimensions. It is logical to think, when suffering from certain personal problem such as irregular sleep patterns, irritability or skipping meals, that this is connected to group problems such as relationship problems and everything is related to, for example, missing deadlines.

One should not lose sight of the moment of technological intensification due to the imposed social isolation to prevent the contagion of COVID-19, and that users who invest greater amounts of time on the Internet more frequently present a connection behavior with a high degree of excitement when on the Internet, loss of control over connection behavior, changes in health habits and interference at a social, family, academic or work level [72]. In a structured way, this work has yielded results on the three levels of consequences. If we focus on individual consequences, our study finds a positive correlation between global technostress and emotional, personal and eating problems (this being the order from highest to lowest correlation). Therefore, many of the relationships reported in the literature have been verified [36]. It is therefore relevant to note that in this work, a relationship between global technostress is found with problems of feelings of depression, irritability and lack of integration. This is in line with the works that indicated a relationship with emotional problems. Regarding personal issues, this work has verified that there is a positive correlation between global technostress and problems related to neglect of daily activities, increased loneliness, lack of concentration, irregular sleep patterns, avoidance of life problems real, as well as reduced daily hygiene and feeding problems.

The relationship between technostress and eating-related disorders should not be given less importance. A positive correlation has been found between levels of technostress and problems related to skipping meals, eating poorly, not enjoying mealtimes and/or interrupting meals. This may be related to physical health problems which can aggravate the mental health problems associated with technostress. We have also found a positive correlation between technostress and physical and economic damage to the subject complaining of this disease.

Focusing on group consequences, our study finds a positive correlation between global technostress and social, family and privacy problems (this being the order from highest to lowest correlation). Here, the positive influence of global technostress is verified with aspects important for well-being such as the reduction of friends, communication skills and their frequency (all face-to-face), relationship problems and the reduction of quality time with the family, as pointed out [52].

Focusing on the consequences of performance, as the study shows, higher levels of technostress are related to absenteeism, missed deadlines and not achieving objectives, in line with what was suggested by Wang et al. [13] and La Torre et al. [36].

In the second part of the study and, assuming the previous research on individual differences, new archetypes for classifying users, based on their behavior on social networks, have been sought. Furthermore, it has been analyzed whether belonging to one type of user or another may be related to levels of technostress and the potential negative consequences. The motivation was to shed light on whether there is a profile that is more prone to suffering from technostress, or that is more sensitive to suffering some negative consequences, since in that case they would require greater attention to offer them greater prevention and coping measures. In the same way, the idea that there may be more resistant profiles could help graduate the strategies in other more vulnerable groups, thus being able to optimize the results.

In order to optimize the results, prevention and coping strategies would be interesting if they could be focused, instead of being applied in a general way. This could be carried out through the classification of Estrada et al. [62]. In this study, we have found it relevant and coherent to use their way of classifying behavior when using technology. Five different archetypes have been used for this: secure, intimate, escapist, narcissist and discrepancy [63].

Although it has been found that there is no correlation between the archetypes studied and the level of global technostress, we must highlight the positive correlation of archetypes with techno-anxiety, which is the dimension with the greatest relationship with global technostress. Therefore, attention should be paid to this specific extent. Techno-anxiety is an emotional response of fear, apprehension or agitation characterized by high physiological activity and tension. To assess this dimension, the RED measurement instrument has evaluated the feeling of tension and anxiety when working with technologies, doubting oneself when using technologies for fear of making mistakes, fear of thinking that information may be lost due to the incorrect use of technology and feeling uncomfortable, irritable and impatient when working with technology. Focusing on this dimension level of techno-anxiety, the order by archetype from highest to lowest was: discrepancy, escapist, narcissist, secure and intimate. This should focus attention on the discrepancy (as the most affected) and intimate (as the least affected) profiles.

Additionally, it has been shown that the archetypes do have a connection with the three levels of consequences (individual, group and professional). This is important since we detected that the individual profiles are related to the problems that are being minimized or avoided in the three dimensions studied. The relationship is stronger with individual consequences.

Delving into the five user profiles and having verified that they do have a statistically significant relationship with techno-anxiety and with the consequences (at the three levels), it is possible to focus on profiles that we could point to as more sensitive, and profiles that are stronger, or more resistant. This can help to optimize the prevention actions to be implemented, since not all profiles experience technological overload in the same way or feel equally overwhelmed.

For this reason, on the one hand, due attention should be paid to the discrepancy and narcissist profiles. Regarding the discrepancy profile, it has been proven that it is the one that experiences the most techno-anxiety. This profile is held by 12.5%, so it is not very important quantitatively and allows us to focus preventive and coping actions. Along with discrepancy, attention should be paid to the detection of the narcissist profile, since it is the one that experiences the negative effects in the strongest way (individual, group and professional). This profile is held by 8.3% of the participants. These two profiles are therefore the least frequent and most sensitive ones.

On the other hand, secure and intimate really show themselves as very interesting profiles. They experience negative effects in a more gentle way (individual, group and professional) and they are also the ones who suffer the least techno-anxiety. It is of great interest to assess that, as can be seen in the data, the most common archetypes are secure (34.7%) and intimate (31.5%). They are the two most frequent profiles (more than half of the participants would be contained in these two profiles), and they are the most resistant ones.

As technostress is an area of research that will surely acquire even more relevance, given the mental health alert situation around the planet as a consequence of COVID-19, which is redesigning the foundations of family, work and social life, with the greater use and penetration of technological tools [18], our findings can contribute to a greater theoretical and applied knowledge of the phenomenon.

This work has some limitations to overcome in future research. On the one hand, it has focused on an extraordinary moment of forced digital acceleration and should repeat itself in a more stable phase and compare the results. This extraordinary moment, although it provides the desired context of digital intensification, could contaminate some results, considering, for example, a study that shows that 24.9% of university students studied in the initial phase of the pandemic showed symptoms of anxiety (0.9 severe) [73]. On the other hand, analyses related to individual differences by age cannot be carried out since the subjects studied are very homogeneous. The study could be approached in other generations and possible differences both in the levels of technostress and in its repercussion could be addressed. Regarding the typology of Altuwairiqi et al. [63], this work is based on the behavioral self-knowledge of the subjects and their own self-report of the main archetype correspondence, assuming the limitations derived from this as well as the possible archetype combinations (having a primary archetype and a secondary archetype).

Regarding future research, in addition to overcoming the previous limitations, specific studies on techno-anxiety should be addressed (which shows a stronger correlation with the global technostress measure and is the dimension that correlates with the archetypes). It would also be important to carry out studies that delve into techno-skepticism, since it is observed in this study that this dimension of technostress tends to behave differently from the other dimensions.

## 6. Conclusions

On the one hand, we wanted to check whether technostress is related to negative consequences in different dimensions (individual, group and professional) and it was possible to verify that the global technostress measure does have a positive and statistically significant relationship with the three dimensions of consequences studied (individual, group and professional). In addition, it was possible to rank this relationship of technostress (from more to less strong) with the individual, group and professional consequences.

On the other hand, this study aimed to analyze whether there is a relationship between the different levels of consequences. In this study, it was possible to verify something that has been taken for granted when studying the possible negative consequences: the fact that some consequences have an influence on others since they are related to each other in a statistically significant way. Hierarchically (from more to less strong), the order would be the individual–group, individual–professional and group–professional.

In addition, this work tries to determine if there is a relationship between the type of ICT user and technostress. After analyzing the data, it has been verified that the type of user does not have a statistically significant relationship with the level of global technostress. Nonetheless, there is a positive correlation between the archetypes and the techno-anxiety dimension. Those with the discrepancy profile are more likely to suffer from this dimension of technostress, and thus should be the focus of specific strategies for its prevention and coping.

Regarding the investigation of whether there is a relationship between the type of ICT user and the consequences suffered, it has been found that the type of user does have a statistically significant and positive relationship with all three levels of consequences (individual, group and professionals). From the most to least strong, the relationship would be between the type of user and individual consequences, followed by professional and group consequences.

Moreover, in this study, it was discovered that people who have the narcissist profile are the ones who suffer all three dimensions of consequences (individual, group and professional) the most, while users with secure and intimate profiles suffer them in the least intense way. This can be useful for targeting diagnostic and prevention measures.

Focusing on user profiles, the most important conclusions are that the secure and intimate profiles should receive special attention, since they are the ones that suffer the least techno-anxiety (and technostress), and those that experience the least negative consequences. They could be presented as more resistant and desirable profiles. Furthermore, they are the two profiles with the most subjects, which is a very positive fact when planning actions. Along the same lines, it should be noted that the discrepancy and narcissist profiles should also be studied in a special way, since the discrepancy profile is the one that experiences the most techno-anxiety (and technostress), and the narcissist profile is the one that experiences the greatest negative consequences. They could present themselves as profiles that require further attention and intervention. In addition, they are the two profiles with the fewest subjects, which is a positive fact to address their targeting.

## Figures and Tables

**Table 1:** Instrument design.

Questionnaire Design Outline	
1	Gender/Generation/Devices/Programs/Connection
2	Technostress [65] The measurement instrument has been specifically developed using the RED General Questionnaire (or Questionnaire of Resources, Emotions/Experiences and Demands in its general version) that arises as a modification and extension of RED.es, and is applicable to any type of samples, not only occupational but also pre-occupational (for example, university students). It has 16 questions, four of them referring to each dimension of technostress: skepticism (TSK), fatigue (TF), anxiety (TA), and inefficacy (TI).
3	Effects [26] Eight types of problems have been used (emotional, family, personal, social, performance, privacy, damage and food) grouped into three dimensions, justified in the theoretical review: individual effects (emotional, personal, harm and food), group effects (family, social and privacy) and professional effects (performance).
4	User Archetypes [63] secure, intimate, narcissist, discrepancy, escapist

**Table 2:** Descriptive statistics of technostress and its dimensions.

Dimensions	Range	Mean	Standard Deviation	Variance
Techno-Skepticism (TSK)	4.00	2.36	0.77	0.59
Techno-Fatigue (TF)	3.75	2.35	0.84	0.70
Techno-Anxiety (TA)	3.75	2.19	0.88	0.78
Techno-Inefficacy (TI)	3.50	1.95	0.85	0.72
Global Technostress	3.56	2.21	0.70	0.48

**Table 3:** Pearson’s correlations between the dimensions of technostress.

Dimensions	TSK	TF	TA	TI	Global Technostress
Techno-Skepticism (TSK)	1	0.53 **	0.54 **	0.52 **	0.76 **
Techno-Fatigue (TF)	0.53 **	1	0.61 **	0.54 **	0.81 **
Techno-Anxiety (TA)	0.54 **	0.61 **	1.00	0.79 **	0.89 **
Techno-Inefficacy (TI)	0.52 **	0.54 **	0.79 **	1	0.86 **
Global Technostress	0.76 **	0.81 **	0.89 **	0.86 **	1

** The correlation is significant at the 0.01 level (bilateral).

**Table 4:** Descriptive statistics of negative effects.

Effects	Range	Mean	Standard Deviation	Variance
Individual (emotional, personal, harm and food)	3.21	1.93	0.59	0.35
Group (family, social and privacy)	3.43	1.66	0.59	0.35
Professional (performance)	4.00	1.59	0.75	0.56

**Table 5:** Sample composition by archetypes.

Archetype	Frequency	Percentage
Secure	117	34.7
Intimate	106	31.5
Escapist	44	13.1
Narcissist	28	8.3
Discrepancy	42	12.5
Total	337	100

**Table 6:** Outer loading, reliability and AVE.

Constructs	Items	Outer Loading	Cronbach’s Alpha	McDonald’s Omega	Composite Reliability	AVE
TSK	TSK1	0.75	0.82	0.86	0.88	0.65
	TSK2	0.80				
	TSK3	0.84				
	TSK4	0.83				
TF	TF1	0.80	0.82	0.86	0.90	0.69
	TF2	0.79				
	TF3	0.85				
	TF4	0.87				
TA	TA1	0.82	0.87	0.88	0.91	0.73
	TA2	0.88				
	TA3	0.84				
	TA4	0.88				
TI	TI1	0.87	0.89	0.90	0.93	0.77
	TI2	0.90				
	TI3	0.89				
	TI4	0.84				
Emotional	E1	0.73	0.82	0.82	0.89	0.52
	E2	0.79				
	E3	0.71				
	E4	0.71				
	E5	0.77				
	E6	0.60				
Personal	PE1	0.90	0.83	0.85	0.90	0.82
	PE2	0.91				
Harm	H1	0.81	0.77	0.85	0.89	0.67
	H2	0.70				
	H3	0.66				
Food	FO1	0.81	0.70	0.72	0.82	0.70
	FO2	0.82				
	FO3	0.80				
	FO4	0.84				
Family	FA1	0.74	0.82	0.86	0.88	0.65
	FA2	0.92				
Social	S1	0.88	0.89	0.89	0.93	0.82
	S2	0.93				
	S3	0.91				
Privacy	PRI1	0.79	0.70	0.85	0.86	0.75
	PRI2	0.94				
Professional	PRO1	0.89	0.79	0.83	0.88	0.71
	PRO2	0.83				
	PRO3	0.80				

**Table 7:** Pearson’s correlations between technostress and effects.

Dimensions/Effects	Individual	Group	Professional
TSK	0.18 **	0.19 **	0.08
TF	0.33 **	0.31 **	0.20 **
TA	0.35 **	0.31 **	0.17 **
TI	0.33 **	0.31 **	0.17 **
Global Technostress	0.36 **	0.34 **	0.19 **

** The correlation is significant at the 0.01 level (bilateral).

**Table 8:** Pearson’s correlations between effects.

Effects	Individual	Group	Professional
Individual	1	0.74 **	0.66 **
Group	0.74 **	1	0.48 **
Professional	0.66 **	0.48 **	1

** The correlation is significant at the 0.01 level (bilateral).

**Table 9:** SEM path analysis.

Path: A ➔ B	Original Path Coefficient	Mean of Sub-Sample Path Coefficient	*p*	*t*-Value
	(β)			
TSK ➔ Emotional	0.00	0.00	0.0182	0.08
TSK ➔ Personal	0.11	0.11	0.0176	6.17 *
TSK ➔ Harm	0.03	0.03	0.0193	1.68
TSK ➔ Food	0.09	0.09	0.0209	4.41 *
TSK ➔ Family	0.00	0.00	0.0188	0.24
TSK ➔ Social	0.08	0.08	0.0197	4.17 *
TSK ➔ Privacy	0.02	0.02	0.0158	1.42
TSK ➔ Performance	0.10	0.10	0.0188	5.21 *
TF ➔ Emotional	0.17	0.17	0.0192	8.82 *
TF ➔ Personal	0.24	0.24	0.0157	15.20 *
TF ➔ Harm	0.13	0.13	0.0170	7.46 *
TF ➔ Food	0.11	0.11	0.0183	5.90 *
TF ➔ Family	0.18	0.18	0.0202	8.94 *
TF ➔ Social	0.19	0.19	0.0189	10.13 *
TF ➔ Privacy	0.09	0.09	0.0152	5.82 *
TF ➔ Performance	0.19	0.19	0.0187	9.99 *
TA ➔ Emotional	0.31	0.32	0.0260	12.09 *
TA ➔ Personal	0.16	0.16	0.0248	6.73 *
TA ➔ Harm	0.10	0.10	0.0421	2.48 *
TA ➔ Food	0.18	0.18	0.0260	4 *
TA ➔ Family	0.19	0.19	0.0259	7.42 *
TA ➔ Social	0.05	0.05	0.0250	1.83
TA ➔ Privacy	0.15	0.15	0.0215	7.06 *
TA ➔ Performance	0.04	0.04	0.0261	1.43 *
TI ➔ Emotional	0.10	0.10	0.0227	4.32 *
TI ➔ Personal	0.09	0.09	0.0214	4.37 *
TI ➔ Harm	0.28	0.28	0.0333	8.47 *
TI ➔ Food	0.18	0.18	0.0260	6.93 *
TI ➔ Family	0.08	0.08	0.0211	3.76 *
TI ➔ Social	0.21	0.21	0.0229	9.33 *
TI ➔ Privacy	0.27	0.27	0.0248	11.06 *
TI ➔ Performance	0.09	0.10	0.0220	4.30 *

* significant when *p* < 0.05 (based on a Student’s two-tailed test, *t* (499); *t* (0.05; 499) = 1.96).

**Table 10:** Spearman correlations between archetypes and technostress.

Archetype	TSK	TF	TA	TI	Global Technostress
Rho Spearman	0.10	0.04	0.13 *	0.06	0.10

* The correlation is significant at the 0.05 level (bilateral).

**Table 11:** Technostress by user type.

Archetype	TSK	TF	TA	TI	Technostress Average
Discrepancy	2.43	2.54	2.49	2.09	2.39
Escapist	2.47	2.49	2.36	2.14	2.37
Narcissist	2.50	2.32	2.19	1.91	2.23
Intimate	2.30	2.18	2.07	1.80	2.09
Secure	2.30	2.37	2.13	1.97	2.19
Total	2.36	2.35	2.19	1.95	2.21

**Table 12:** Spearman correlations between archetypes and the effects of technostress.

Archetypes	Individual	Group	Professional
Correlation coefficient	0.17 **	0.14 **	0.16 **

** The correlation is significant at the 0.01 level (bilateral).

**Table 13:** Effects by archetype.

Archetype	Individual Average	Group Average	Professional Average
Discrepancy	2.09	1.80	1.61
Escapist	2.10	1.77	1.70
Narcissist	2.29	1.97	2.10
Intimate	1.76	1.56	1.46
Secure	1.88	1.59	1.53
Total	1.93	1.66	1.59

## Data Availability

The dataset generated for this study is available on request to the corresponding author.
